# Onshore preparedness for hazardous chemical marine vessel accidents: A case study

**DOI:** 10.4102/jamba.v8i1.246

**Published:** 2016-09-20

**Authors:** Faisel T. Illiyas, Keshav Mohan

**Affiliations:** 1 Institute of Land and Disaster Management, Government of Kerala, India

## Abstract

Hazardous and noxious substances (HNS) are widely transported in marine vessels to reach every part of the world. Bulk transportation of hazardous chemicals is carried out in tank container–carrying cargo ships or in designed vessels. Ensuring the safety of HNS containers during maritime transportation is critically important as the accidental release of any substance may be lethal to the on-board crew and marine environment. A general assumption in maritime accidents in open ocean is that it will not create any danger to the coastal population. The case study discussed in this article throws light on the dangers latent in maritime HNS accidents. An accident involving an HNS-carrying marine vessel in the Arabian Sea near the coast of Yemen became a safety issue to the coastal people of Kasargod District of Kerala, India. The ship carried more than 4000 containers, which were lost to the sea in the accident. Six HNS tank containers were carried by the waves and shored at the populated coast of Kasargod, more than 650 nautical miles east from the accident spot. The unanticipated sighting of tank containers in the coast and the response of the administration to the incident, the hurdles faced by the district administration in handling the case, the need for engaging national agencies and lessons learned from the incident are discussed in the article. This case study has proven that accidents in the open ocean have the potential to put the coastal areas at risk if the on-board cargo contains hazardous chemicals. Littoral nations, especially those close to the international waterlines, must include hazardous chemical spills to their oil spill contingency plans.

## Introduction

Shipping has evolved as one of the major transportation industries in the world. Global economy depends on shipping for cost-effective transportation of bulk cargos over long distances. The safety and security of maritime transport is often governed by international regulations followed by the maritime nations (International Maritime Organisation [IMO] 2012). Oil spill is considered as one of the major economic, environmental and hazardous challenges faced by the shipping industry. The common oil spills in ports and oil terminals occur during routine operations such as loading, discharging and bunkering and along transportation routes during collisions and groundings. Preparedness and response measures adopted by oil or shipping industries and the governments of maritime member countries have reduced oil spill incidents in the late 2000s to one fifth of that during the 1970s (Purnell 2009). This is reiterated by the annual tanker spill analysis by International Tanker Owners Pollution Federation (ITOPF) (ITOPF 2014). Maritime transport of chemicals has also increased during the same period but without adequate preparedness measures as are found in the case of oil spills.

Chemical industries have emerged as the major player in global economy, which requires large flows of goods from production sites to consumption sites. Growth of chemical industries led to significant increase in the proportion of maritime transportation carrying dangerous and hazardous chemicals. Of the 37 million chemicals used by the world population, 2000 are regularly transported by sea. The volumes shipped are currently on the rise, with maritime chemical transport having more than tripled in the past 20 years. About 10% – 15% of the cargos transported over water are dangerous goods of various forms (IMO 1996; Purnell 2009). In 2013, 94 large ships were lost worldwide, and cargo ships account for a third of these losses (Allianz Global Corporate and Specialty [AGCS] 2014).

Chemical spill occurs at relatively lower frequency than the oil spills, but its consequences will be more disastrous (ITOPF 2014). The risks have become increasingly acute, in particular, because of the growing number of ultra large ships together with the high intensity imposed by global market pressure. The threat of a chemical spill at sea concerns many public and private interest groups as the pollution caused may often be invisible and may appear difficult to manage. Risk of marine accidents involving dangerous goods is not confined to the risk to the life of on-board crew; it further causes serious damage to the marine environment and coastal habitation. Chemical risk caused by marine vessel accidents needs to be investigated in detail to develop effective risk management strategies and for evolving prompt response mechanisms in international waters and coastlines.

## Hazardous and noxious substances

The protocol on preparedness, response and co-operation to pollution incidents by hazardous and noxious substances defines hazardous and noxious substances (HNS) as:

any substance other than oil which, if introduced into the marine environment, is likely to be hazardous to human health, to harm living resources and marine life, to damage amenities or to interfere with other legitimate uses of the sea. (IMO 2007:2)

A substance is classified as hazardous or noxious when the chemical transported has one or more of the following properties: flammability, exclusivity, toxicity, corrosivity, reactivity or radioactivity or is infectious (Purnell 2009). Chemicals having different physical or chemical properties behave differently when they are spilt into water. Based on the physical and chemical properties, the Standard European Behaviour Classification system theoretically classifies chemical substances as gases, evaporators, floaters, dissolvers and sinkers. Chemical substances normally have more than one behaviour rather than a single property, and their behaviour is influenced by environmental processes such as wind, waves and current (CADRE 2012). Information on the behaviour of HNS spilt must be gathered by the emergency response agencies in order to design response actions with safety considerations.

## Transportation of hazardous and noxious substances

**FIGURE 1:**
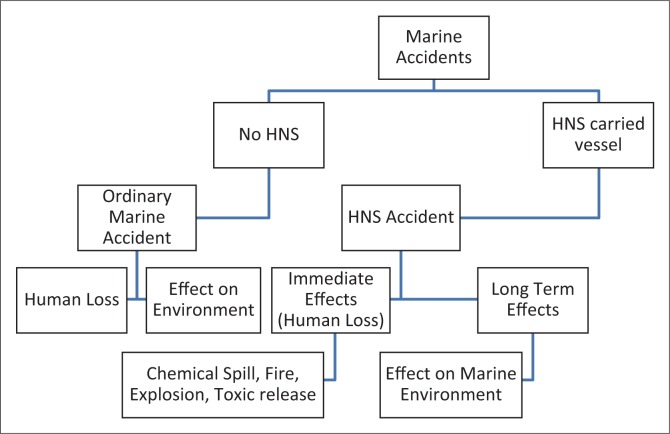
Effect of marine accidents involving vessels carrying hazardous and noxious substances.

HNS are transported in both bulk and packaged form. In bulk cargo, materials of homogeneous nature are transported in specific containers. Packaged materials are carried as semi-manufactured and manufactured commodities in consignments (Mullai 2006). The International Maritime Dangerous Goods (IMDG) Code has been developed by the IMO in accordance with the resolution of International Convention for the Safety of Life at Sea 1960, which sets out minimum requirements or standards for the transport of dangerous goods by all modes of transport. IMDG code is a uniform international code for the transport of dangerous goods by sea. Based on the hazardous nature of the chemicals transported, it classifies dangerous goods into nine major classes. They are (1) explosives, (2) gases, (3) flammable liquids, (4) flammable solids, (5) oxidisers and organic peroxides, (6) toxic and infectious substances, (7) radioactive material, (8) corrosive substances and (9) miscellaneous dangerous substances (IMO 2015). HNS carriers involved in accidents often pose threat of chemical hazards, which is difficult to manage in the marine environment. Marine accidents such as grounding, collision, engine failure, etc., may lead to chemical fire, explosion, spillage or toxic release causing death and severe deterioration of the marine environment. Figure 1 shows the effect of marine accidents involving HNS-carrying vessels.

If vessels carrying more than one HNS materials meet with an accident, it may lead to mixing of chemicals, initiating chemical reactions. The reactive nature of the substances makes it more hazardous. To anticipate such a hazardous incident, the ship crew must have adequate understanding about the hazardous and reactive nature of the substances they are carrying. The risk of marine accidents involving dangerous goods depends on the physical, chemical and hazardous properties and the quantity of substances.

## Hazardous and noxious substances accident in Arabian Sea: A case study

**FIGURE 2:**
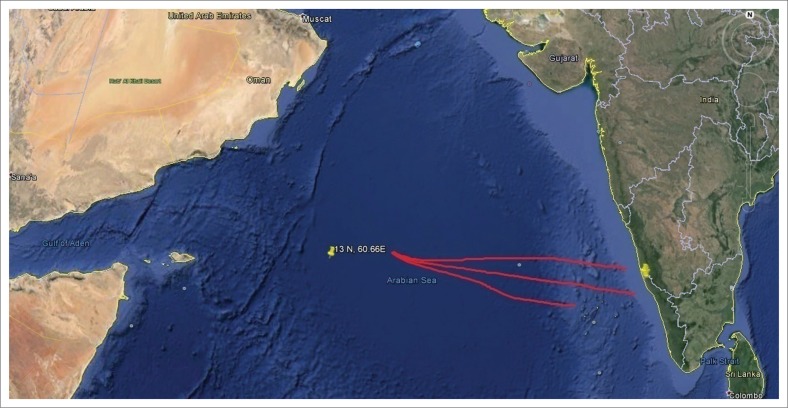
Ship accident location in Arabian Sea. Stranded containers moved to the western coast of India and washed ashore in Kasargod coast, Kerala, India.

**FIGURE 3:**
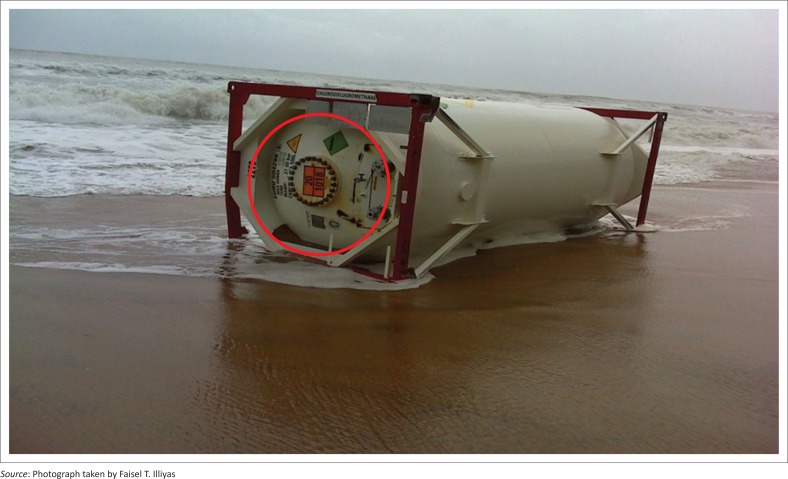
Container at Jenma beach. Emergency Information Panel including coloured symbol can be seen inside the red circle.

**FIGURE 4:**
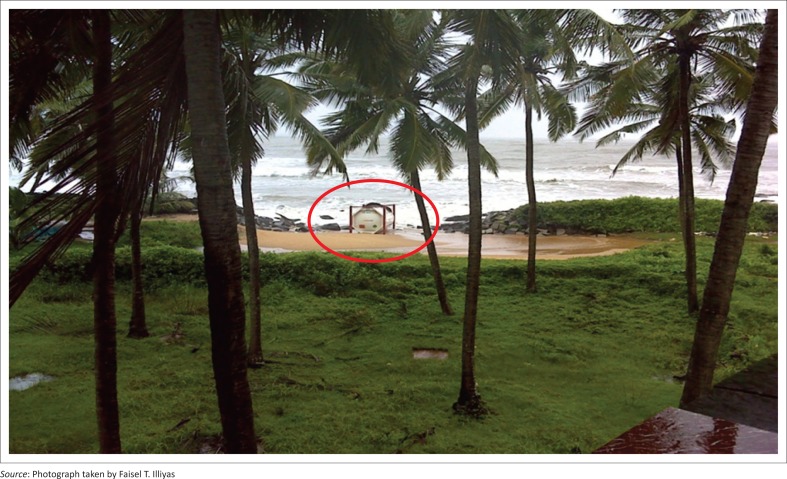
Container at Kottikulam beach found in between the damaged sea wall.

**FIGURE 5:**
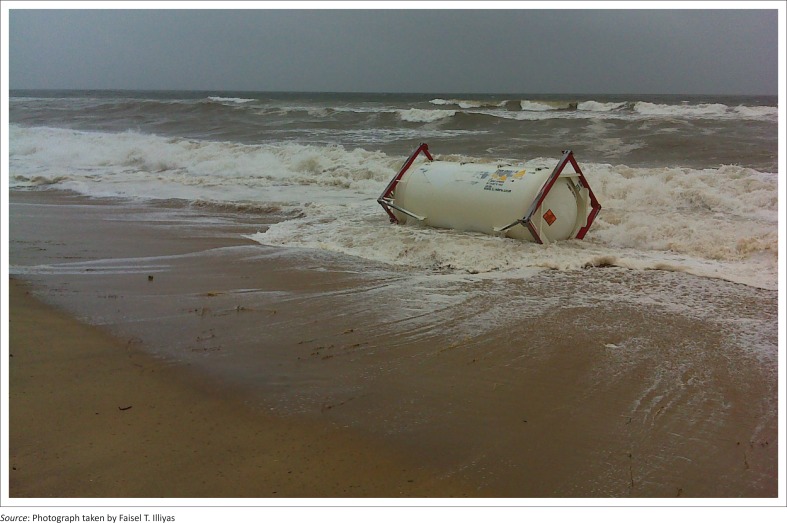
Container at Mogral beach moving by the wave force.

**FIGURE 6:**
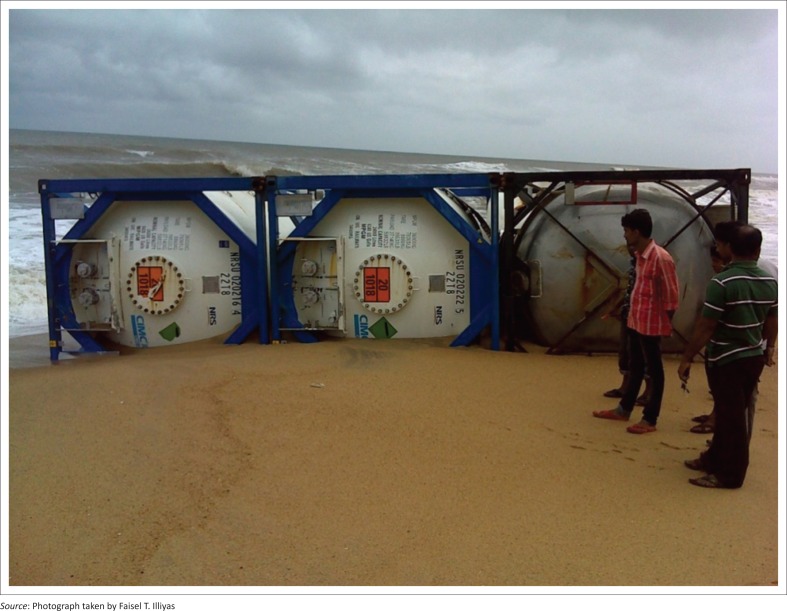
Three containers found together at Berikka beach.

The container ship ‘MOL COMFORT’ operated by Tokyo-based Mitsui O.S.K. had met with a marine accident during its sail from Singapore to Jeddah in June 2013. The ship carried 4382 containers of different substances. On 17 June 2013, in the Arabian Sea near the coast of Yemen, the vessel collapsed and fractured into two, fore and aft (Committee on Large Container Ship Safety [CLCSS] 2013). The fore part of MOL Comfort was towed by a tug vessel of the salvage company, after the aft part of MOL Comfort sank into deep sea. A fire broke out from the rear end of MOL Comfort on 06 July 2013. A salvage vessel of Indian Coast Guard *Samudra Prahari* with external fire fighting system and other salvage troops responded to the fire aboard the fore part of MOL Comfort. On-board containers of the wrecked ship were lost overboard or damaged during the accident. It was reported on the early morning of 24 July 2013 that large containers were washed ashore on the western coast of India at various locations of Kasargod District, in the state of Kerala, in India. The location of the accident and the container movement direction is given in Figure 2. On noticing the containers at the sea shore, the local residents reported the unusual incident to the nearby police stations. The residents of the area had never before witnessed such an incident at the shore. This got wide public and media attention. Containers were also found in four distant coastal locations of Kasargod (Figures 3–6). Along with the containers, other products such as refrigerators, thermal flasks, footballs, beer cases, et cetera, were also found ashore. The articles other than the containers were taken away by the local people.

## Administrative response

Several stories popped up about the containers. Rumours spread among the public that the containers had toxic gas inside and may explode. The district administration responded immediately and deployed the police on the site to safeguard the containers from any further damage. The incident was brought to the notice of the Indian Coast Guard, Cochin Port Trust and the Director General of Shipping so that the containers could be moved to a safer place from the populated coastal area. During the initial phase, the responding departments had no information about the type of chemicals inside the containers. The district administration assigned an expert team comprising a forensic expert, representatives from the ship insurance agency, police, coast guard and Disaster Management Department to inspect the containers and advise the administration on the action to be taken, in order to allay the fear of the public. The investigation team inspected six containers, which washed ashore in Jenma beach, Kottikulam beach, Mogral beach and Berikka beach in Kasargod.

## Hazardous and noxious substances in containers

One method of identifying chemicals in a cargo container is to read the Emergency Information Panel (EIP) on the container. EIP contains the technical name of the chemical carried, hazardous nature symbol, IMDG code, container capacity, consignment number, et cetera. An expert committee on verification of the container EIP inferred that chlorodifluoromethane was the chemical within the containers found in Jenma, Kottikulam and Mogral beaches. Chlorodifluoromethane is a colourless noncombustible gas with an ethereal odour. It is shipped as a liquefied gas under its own vapour pressure. It can displace the air on release to the environment and cause asphyxia. If the chemical container is exposed to prolonged heat or fire, it may rupture violently and rocket (National Oceanic and Atmospheric Administration [NOAA] 2013a).

Tank containers used for transporting chemical substances have gauges to monitor safety parameters. The gauges in the containers washed ashore were out of order, which limited the capability of the investigation team to figure out the quantity of chemicals. Containers were buoyant and moving with the waves. The container at Kottikulam beach was seen in between the rocks of the damaged sea wall. Considering the risk of the container floating and hitting on rocks, it was towed to nearby trees using steel ropes that avoided the chance of damage and leakage.

Three containers together with the anchoring cases were seen in Berikka beach. Of the three containers, two contained chlorodifluoromethane as identified from the EIP. The third container was found to be severely damaged, and its emergency identification marks were not in position. On inspection, the cargo number engraved on the container was noted and verified against the cargo manifest. The chemical carried in the container was alkyl amine, which is a flammable or combustible material, liable to be ignited by heat, sparks or flames. On leakage, vapours may form explosive mixtures with air. It is also corrosive or irritant in nature (NOAA 2013b).

## Crisis management

A crisis management meeting was convened by the District Collector to find suitable solutions for disposal of the container. First priority was to shift the container from the populated vulnerable beach to a safer place. Assistance was sought from nearby Ports and the Director General of shipping as shifting required towing vessels, advanced equipment and experts. Ship wreckage had resulted in the floating of large number of containers in the open ocean. It was feared that containers at sea may cause accidents to fishing boats as the fishing season in the Kerala coast was to commence in August 2013, just a month later. Considering all the above facts, the matter was referred to the National Disaster Management Authority and Ministry of Defense, Government of India, for technical support. Precautions and actions taken by the administration to ensure public safety were communicated to local people through media, which helped to assuage the fears and control the situation at the grass-roots level. In the crisis management meeting, the insurance agency of the wrecked ship agreed to acquire the containers and after 3 months they managed to remove the containers from the Kasargod beach.

## Lessons learned

Maritime accident that occurred near the coast of Yemen turned to be a chemical hazard to the coastal people of Kasargod in Kerala, India. The case gives an important lesson that accidents involving marine vessels carrying HNS in the open ocean pose a potential threat, transforming into and progressing into an onshore hazard to coastal areas. The state administration has limited experience in dealing with maritime accidents involving hazardous cargo. A major constraint of the state administration in responding to a maritime accident is the incomplete information about the incident and the chemical substances carried. Moreover, the states may not have sufficient resources such as specialised equipment, experienced manpower, response vessels, etc., to deal with such substances. Hence, maritime accidents with HNS substances may be directly handled by the national agencies with support from state government departments and district administration. Involvement of coast guard, Navy and Shipping Department and nearby ports is very crucial in dealing with the maritime HNS accidents.

India has national- and regional-level oil spill contingency plans on board. The Indian Coast Guard acts as the central agency for coordination and implementation of oil spill contingency plans (Indian Coast Guard 2010). But the contingency actions for HNS accidents were not addressed in any of the national or regional plans. Revision of national and regional plans by including the components of HNS will be the appropriate way to address maritime HNS accidents. States sharing coastal boundaries shall prepare state-level chemical spill response plan for which assistance from major industries may be sought for preparedness and response planning.

The agencies involved in handling the HNS accidents should be aware of the hazardous nature and potential reaction of the chemicals carried. A rapid and precise risk assessment should be performed before the cargos are moved or disposed. Indian Coast Guard being the central agency for coordination of maritime accident response in India must have officers competently trained in handling HNS. The protocols suggested by Qiuhui (2011), suitably modified to local conditions, can be used as a framework for dealing with spills and beaching of containers.

## Conclusion

Shipping hazardous chemicals over international waters has increased considerably over the past 20 years. Many of the chemicals transported in cargo ships are highly hazardous and more toxic than oils. Chemical spills at sea may create multifaceted impacts to humans more than environmental deterioration. Effect of hazards and noxious substance spills in ocean depends on the nature of chemical spilled, its quantity and location of the spill. Chemical spills near the coast or harbours may pose more safety concerns to the coastal population and tourism sector. Preparedness to manage HNS spills are not up to the mark as compared to oil spills. The case study of MOL Comfort accident shows that ship accidents at open ocean carrying HNS containers may turn into a serious safety issue to the coastal population. Water currents can take the wreckage of a ship accident to any coastal area. If it includes chemical containers, the administration has to be involved to deal with the safety of the public. Unavailability of reliable information and lack of expertise limit the local administration from immediate response to any onshore incidents. The coast guard, coastal police and harbour departments should come forward to help the local administration to deal with any onshore HNS accidents.

## References

[CIT0001] Allianz Global Corporate and Specialty (AGCS), 2014, *Safety and shipping review 2014, An annual review of trends and developments in shipping losses and safety*, viewed 27 April 2015, from http://www.agcs.allianz.com/assets/PDFs/Reports/Shipping-Review-2014.pdf

[CIT0002] CADRE, 2012, *Understanding chemical pollution at Sea, Learning guide*, Cedre, Transport Canada, Brest, viewed 15 January 2015, from http://www.cedre.fr

[CIT0003] Committee on Large Container Ship Safety (CLCSS), 2013, *Interim report of committee on large container ship safety*, English Version, Japan, viewed 11 March 2015, from http://www.mlit.go.jp/common/001029660.pdf

[CIT0004] Indian Coast Guard, 2010, *National oil spill disaster contingency plan*, viewed 08 March 2015, from http://www.indiancoastguard.nic.in/Indiancoastguard/NOSDCP/NOSDCP%20Incidents.htm

[CIT0005] International Maritime Organisation (IMO), 1996, *Code of the investigation of marine casualties and incidents*, IMO Document, FSI 5/10/2, DEC 1996, International Maritime Organisation, London, UK.

[CIT0006] International Maritime Organisation (IMO), 2007, *Protocol on preparedness, response and co-operation to pollution incidents by Hazardous and Noxious Substances*, 2000 (OPRC-HNS Protocol), International Maritime Organisation, viewed 07 December 2013, from http://www.imo.org/About/Conventions/ListOfConventions/Pages/Protocol-on-Preparedness,-Response-and-Co-operation-to-pollution-Incidents-by-Hazardous-and-Noxious-Substances-%28OPRC-HNS-Pr.aspx

[CIT0007] International Maritime Organisation (IMO), 2012, *International shipping facts and figures – Information resources on trade, safety, security, environment*, Maritime Knowledge Centre, London, UK.

[CIT0008] International Maritime Organisation (IMO), 2015, *International Maritime Dangerous Goods (IMDG) Code*, International Maritime Organisation, viewed 17 August 2015, from http://www.imo.org/blast/mainframe.asp?topic_id=158

[CIT0009] International Tanker Owners Pollution Federation (ITOPF), 2014, *Hazardous and noxious substances*, viewed 24 March 2015, from http://www.itopf.com/knowledge-resources/documents-guides/hazardous-and-noxious-substances-hns/

[CIT0010] Mullai A, 2006, *Maritime transport and risks of packaged dangerous goods*, DaGoB, Turku, Finland, ISBN 951-564-391-0.

[CIT0011] National Oceanic and Atmospheric Administration (NOAA), 2013a, *CAMEO Chemicals-ChloroDifluoro Methane*, viewed 02 June 2014, from http://cameochemicals.noaa.gov/chemical/2889

[CIT0012] National Oceanic and Atmospheric Administration (NOAA), 2013b, *CAMEO Chemicals-alkylamines or polyalkylamines, [liquid, flammable, corrosive]*, viewed 02 June 2014, from http://cameochemicals.noaa.gov/chemical/14562

[CIT0013] Purnell K, 2009, ‘Are HNS Spills more dangerous than oil spills?’, A White Paper for the Inter Spill Conference & the 4th IMO R& D Forum, Marseille, May 2009, pp. 4–5.

[CIT0014] Qiuhui Q, 2011, ‘Safety issues at spills’, in Fingas M. (ed.), *Oil spill science and technology prevention, response, and cleanup*, p. 1189, Gulf Professional Publishing, Burlington, NJ.

